# Massive duodenal ulcer bleeding due to the ruptured hepatic artery pseudoaneurysm after living donor liver transplantation

**DOI:** 10.1186/s40792-022-01558-8

**Published:** 2022-10-18

**Authors:** Masashi Kadohisa, Yukihiro Inomata, Masataka Sakisaka, Yasuhiko Sugawara, Taizo Hibi

**Affiliations:** 1 Department of Transplantation and Pediatric Surgery, Kumamoto University, Honjo 1-1-1, Chuo-Ku, Kumamoto-Shi, Kumamoto 860-8556 Japan; 2 Kumamotorousai Hospital, Kumamoto, Japan; 3 Sakisaka Hospital, Kumamoto, Japan

**Keywords:** Hepatic artery pseudoaneurysm, Gastrointestinal bleeding, Duodenal ulcer, Living donor liver transplantation

## Abstract

**Background:**

The rupture of a hepatic artery pseudoaneurysm (HAP) is a rare but lethal complication after living donor liver transplantation (LDLT) and often manifests as acute gastrointestinal bleeding.

**Case presentation:**

This report describes three patients who experienced HAP after LDLT. These patients initially presented with active bleeding of a duodenal ulcer (DU) in the duodenal bulb, followed by diagnosis of the ruptured HAP by angiography. None of the patients had evidence of an active intra-abdominal infection or bile leakage preceding the rupture of HAP. All patients were initially treated by transcatheter arterial coil embolization (TAE). In all cases, TAE was successful for hemostasis but resulted in complete obstruction of the arterial inflow to the graft. Arterial revascularization by surgical reconstruction using the autologous arterial graft in one case and re-LDLT in another one was successfully performed. The other one succumbed to sepsis caused by later liver abscesses.

**Conclusion:**

This is the first detailed case series of massive DU bleeding as a warning signal of ruptured HAP after LDLT. HAP should be included in the differential diagnosis when an LDLT recipient presents with gastrointestinal bleeding.

## Background

Hepatic artery pseudoaneurysm (HAP) is a rare but critical complication after liver transplantation, with an incidence of 0.27–2.0% [1–9]. Although the recognition of HAP before rupture is associated with a successful outcome, HAP detection after the rupture is associated with a high mortality rate. The rupture often manifests as acute gastrointestinal bleeding or intra-abdominal bleeding [1–9]. The gastrointestinal bleeding is more common than the peritoneal bleeding [2, 3, 5–9]. HAP usually occurs within the first few months following liver transplantation and is commonly reported to be associated with peritoneal infection, technical difficulties during arterial anastomosis, and biliary leak [1–4, 7].

Peptic ulcer disease (PUD) including duodenal ulcer (DU) can cause critical outcomes when hemorrhage and perforation occur [10, 11]. Generally, non-steroidal anti-inflammatory drugs and *Helicobacter pylori* are the two major risk factors for PUD [12]. DU bleeding associated with ruptured HAP after LDLT has rarely been described in the field of liver transplantation [13–16].

Between December 1998 and December 2020, 557 consecutive patients underwent living donor liver transplantation (LDLT) at Kumamoto University Hospital. Of these, three patients (0.5%) experienced ruptured HAP following LDLT. In this paper, we report in detail the clinical courses of the ruptured HAP associated with DU with special focus on the disease mechanism.

## Case presentation

The clinical features and outcomes of the three cases are summarized in Table 1.

**Table 1:** The clinical features and outcomes of the three cases described in our study involving hepatic artery pseudoaneurysm rupture

Case	Age	Sex	Primary disease	Graft type	Arterial anastomosis		Biliary anastomosis	Complication before HAP	Interval after LDLT (months)	Primary symptom	Treatment	Outcome
					Recipient	Donor						
1	49	Male	FAP	Right	RHA	RHA	Duct-to-duct	Acute cellular rejection	1	Hematemesis	TAE × 2 → revascularization	Alive
2	59	Male	HBV HCC	Posterior	RHA	RPHA	Duct-to-duct	Nothing	2	Tarry stool	TAE	Dead
3	62	Male	IPH	Right	RHA	RHA	Duct-to-duct	Biliary stenosis	19	Hematemesis	TAE → re-LDLT	Alive

*HAP* hepatic artery pseudoaneurysm, *FAP* familial amyloid polyneuropathy, *HBV* hepatitis B virus, *HCC* hepatocellular carcinoma, *IPH* idiopathic portal hypertension, *RHA* right hepatic artery, *RPHA* right posterior hepatic artery, *TAE* transcatheter arterial coil embolization, *LDLT* living donor liver transplantation

### Case 1

This case has already been reported previously [17]. In brief, a 49-year-old man underwent LDLT for familial amyloid polyneuropathy using a right lobe graft. Arterial reconstruction was performed with end-to-end anastomosis between the recipient’s right hepatic artery (RHA) (3.5 mm in diameter) and the RHA of the graft (3.0 mm in diameter). Postoperative Doppler ultrasound had showed good arterial flow. No microorganism from abdominal drain fluid and no inflammatory response to abdominal infection had been confirmed. He repeatedly bled profusely from a duodenal ulcer. Therefore, an emergency angiography was performed, which revealed excessive bleeding into the duodenum from the pseudoaneurysm located near the anastomotic site of the hepatic artery on postoperative day (POD) 41. Transcatheter arterial coil embolization (TAE) was performed to treat the bleeding from the aneurysm. Hemostasis was successfully achieved. However, the arterial supply to the liver was completely obstructed. Subsequently, surgical repair of the hepatic artery was performed on POD 56 using an autologous inferior mesenteric artery graft, which was interposed between the graft hepatic artery and the recipient’s gastroduodenal artery. He had an uneventful postoperative course.

### Case 2

**Fig. 1:**
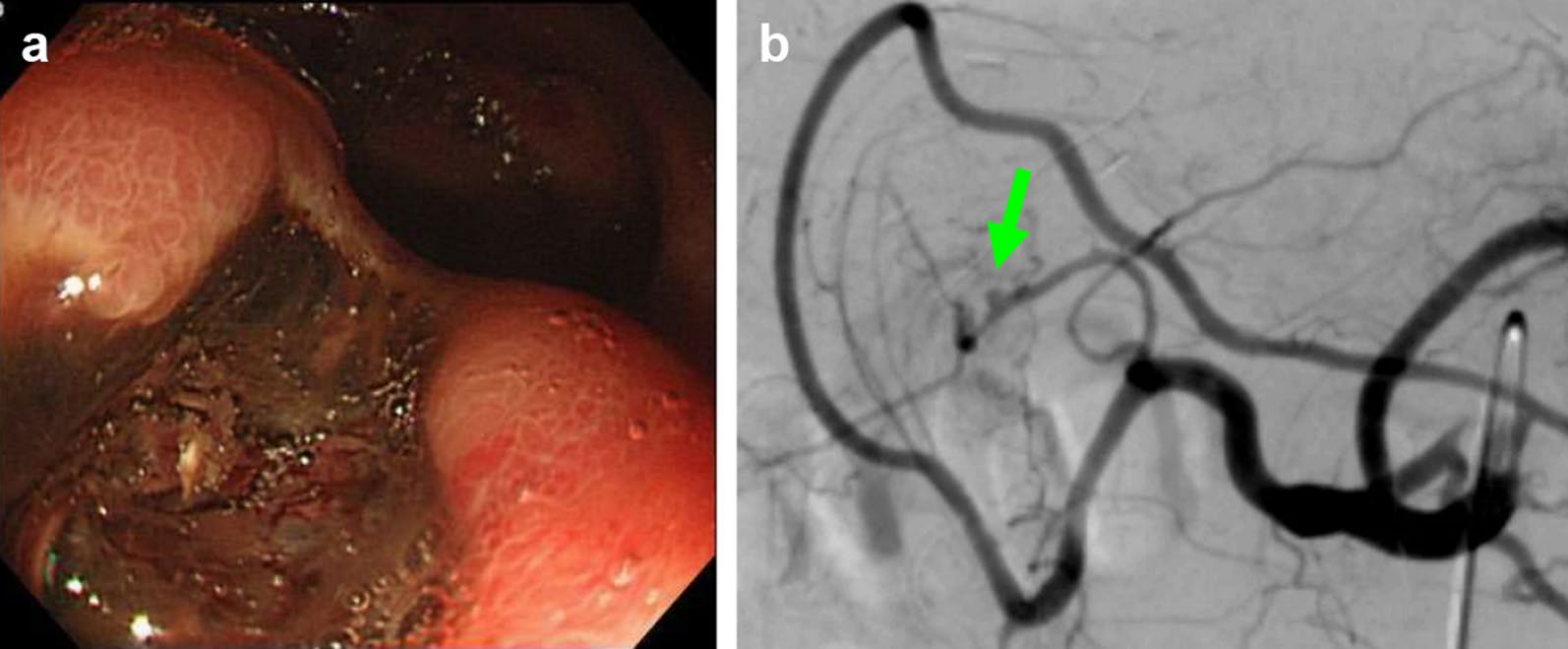
**a** In case 2, an emergent endoscopic examination revealed a deep ulcer in the posterior wall of the duodenal bulb. **b** Angiography revealed a HAP (arrow) proximal to the anastomotic site of the hepatic artery

A 59-year-old man with hepatitis B-related liver cirrhosis and hepatocellular carcinoma underwent LDLT with a right posterior lobe graft from his son. Arterial reconstruction was performed with end-to-end anastomosis between the recipient’s RHA (3.5 mm in diameter) and the posterior branch of the hepatic artery of the graft (2.0 mm in diameter). Postoperative Doppler ultrasound had showed good arterial flow. No microorganism from abdominal drain fluid and no inflammatory response to abdominal infection had been confirmed. Although he experienced dysgeusia and decreased appetite, he was discharged on POD 52. However, he presented with tarry stool and anemia on POD 57. An urgent endoscopic examination revealed a deep ulcer in the posterior wall of the duodenal bulb (Fig. 1a). Endoscopic treatment was successful, resulting in complete hemostasis. On POD 68, angiography was performed for the recurrent bleeding, and it revealed a HAP proximal to the anastomotic site of the hepatic artery (Fig. 1b). TAE was performed, which resulted in complete obstruction of the arterial inflow to the graft. Initially, liver function was stable. The patient was monitored with the expectation of collateral formation. However, within 2 months, multiple liver abscesses and graft dysfunction occurred. No donor was available for retransplantation. Eventually, he died 177 days after the TAE.

### Case 3

**Fig. 2:**
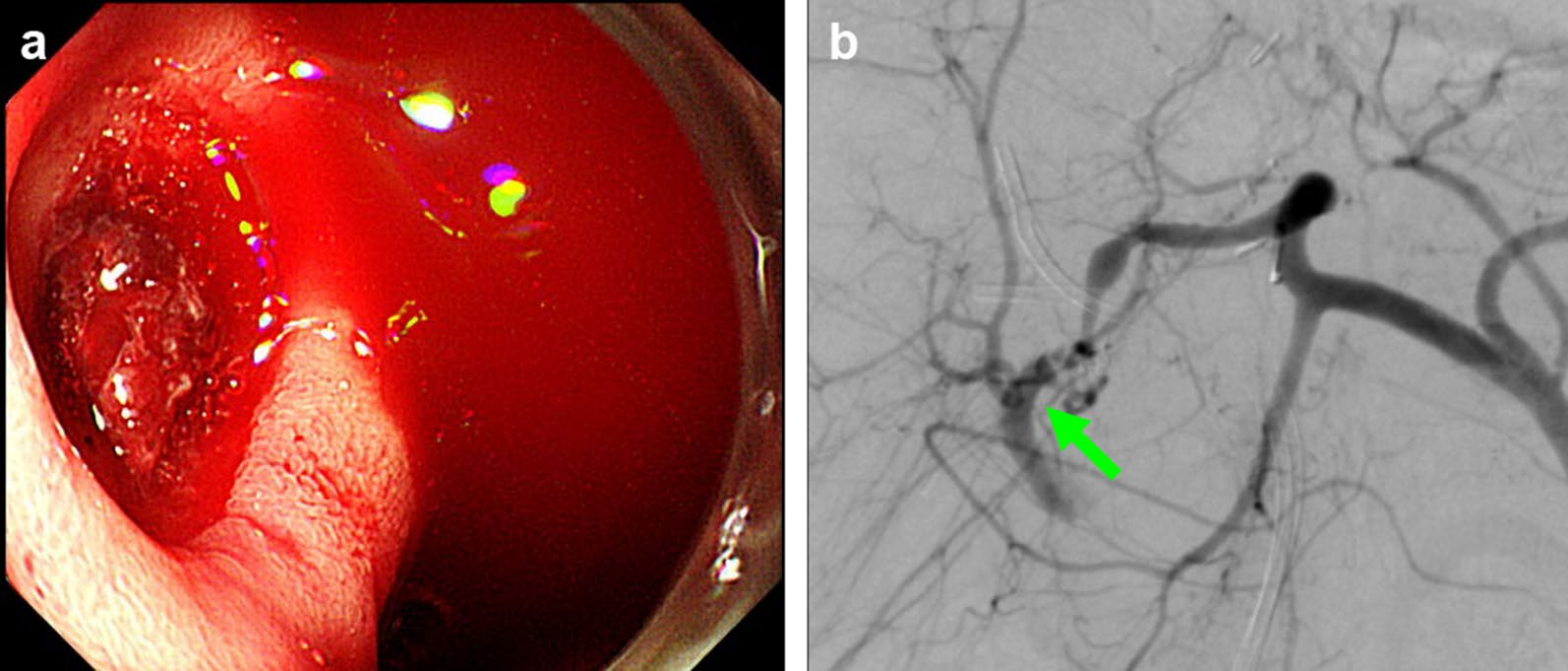
**a** In case 3, an emergent endoscopic examination revealed active bleeding from a duodenal ulcer in the anterior wall of the duodenal bulb. **b** Angiography revealed the bleeding point (arrow) near the bifurcation of the anterior and posterior branches of the RHA of the graft

A 62-year-old man with idiopathic portal hypertension underwent LDLT with a right lobe graft from his wife. Arterial reconstruction was performed with end-to-end anastomosis between the recipient’s RHA (6.0 mm in diameter) and the hepatic artery of the graft (5.0 mm in diameter). The bile duct had been reconstructed in a duct-to-duct fashion, between the right hepatic duct of the graft and the stump of the right hepatic duct of the recipient. Postoperative Doppler ultrasound had showed good arterial flow. No microorganism from abdominal drain fluid and no inflammatory response to abdominal infection had been confirmed. Afterwards, he developed an anastomotic stenosis of the bile duct. Although endoscopic retrograde cholangiopancreatography and biliary stenting were performed, recurrent cholangitis occurred. During postoperative month 19, he was readmitted to the hospital because of fever and elevated liver function. On day 3 after admission, he suddenly developed massive hematemesis. An urgent endoscopic examination revealed active bleeding from a duodenal ulcer in the anterior wall of the duodenal bulb (Fig. 2a). Endoscopic hemostasis was not successful, and an urgent angiography was performed. The bleeding point was identified near the bifurcation of the anterior and posterior branches of the RHA of the graft (Fig. 2b). Due to the location of the bleeding site, surgical reconstruction was judged to be impossible. TAE was performed, which resulted in the complete obstruction of the arterial inflow to the liver. During the subsequent weeks, the patient’s condition was complicated by occurrence of multiple liver abscesses and high fever similar to that observed in case 2. Conservative treatments using antibiotics and percutaneous drainage of the abscess could not stabilize his general condition. Re-LDLT was performed 33 days after the coil embolization, with left lobe graft taken from his son. Arterial reconstruction was performed with end-to-end anastomosis between the recipient’s right gastroepiploic artery and the donor’s left hepatic artery. He had an uneventful postoperative course after the re-LDLT.

## Discussion

In this paper, we reported in detail three cases of ruptured HAP associated with massive DU bleeding. Although ruptured HAP after liver transplantation has been sporadically reported, very few studies have focused on the relationship between ruptured HAP and DU bleeding [13–16].

Out of the 557 patients who underwent LDLTs, we observed 3 cases of HAP (incidence: 0.5%). This was within the reported range (0.27–2.0%) for the incidence of HAP [1–9]. A few studies reported that the incidence of HAP was 1.1–5.5% [4–6] and that LDLT did not result in a specific risk factor for HAP [4]. Interestingly, all the reported cases of HAP in LDLT, including the cases in our study, involved a right-side graft [4–6, 13, 14, 18, 19].

Intra-abdominal infection, bile leakage, bilioenteric anastomosis, and technical difficulties during arterial anastomosis were reported as risk factors associated with HAP following LDLT [1–4, 7]. In our series, duct-to-duct anastomosis was performed in all the three patients, and neither intra-abdominal infection nor bile leakage occurred. In all three patients, no intimal dissection was evident at the time of hepatic artery reconstruction and the anastomoses were uneventful. Only the patient in case 3 had biliary stenosis. Although a biliary stent was placed endoscopically, recurrent cholangitis was not well controlled. A few reports suggested a relationship between biliary stent and HAP [9, 20, 21].

HAPs after LDLT were often recognized as gastrointestinal bleeding or intra-abdominal bleeding [1–9]. The most common symptom is acute abdominal pain with shock [1–9]. In our series, all three patients developed active ulcer bleeding in the duodenal bulb despite being on proton pump inhibitor. When recipients present with hematemesis or tarry stool, and endoscopic examination reveals DU, HAP should be considered as a differential diagnosis.

We must consider the proper approach to HAP, for example immediate revascularization of the hepatic artery including surgery and stent placement or re transplantation, even after successful hemostasis by endoscopic treatment or TAE. A number of recent studies have reported the usefulness of stent placement [4, 5, 9, 13, 22–24]. Nowadays, the standard treatment for HAP should be the stent placement. In our cases involving rupture, stent placement was judged inadequate to achieve hemostasis therefore we selected TAE first. However, it may be dependent on experiences and ability of each center.

We have considered that tortuosity of the vessels and the site of HAP were the deciding factor for performing revascularization including stent placement. Tracking of stents would be difficult through tortuous vessels, especially after arterial vascular reconstruction in LDLT. Moreover, the site of HAP was the key to place stent with small interstices, for example, in case 3, the bleeding point was near the bifurcation of the anterior and posterior branches of the right hepatic artery. We decided it was impossible to place the stent. If we attempted the re-do, we had to prepare two orifices of the HA in the recipient side, and it was considered to be not realistic.

In all patients described in our study, DU bleeding occurred after right lobe LDLT. The initial source of HAP is usually the site of arterial anastomosis. The site of hepatic artery anastomosis after right lobe LDLT is close to the duodenal bulb. Therefore, the anastomotic site and the duodenal bulb may have a direct contact with each other. DU may cause an inflammatory change in the hepatic artery close to the duodenum. Moreover, the local inflammation causes the fistula formation between pseudoaneurysm and duodenum. Although it is difficult to confirm whether the HAP or DU occurred first, at least cases 1 and 2, penetration of DU may have preceded the formation of HAP, because there were no predisposing factors. In case 3, although there was no evidence of intra-abdominal infection or bile leakage, recurrent cholangitis may have provoked localized infection near the arterial anastomosis, resulting in HAP development.

## Conclusions

HAP is a rare but lethal complication after liver transplantation. It is often recognized as acute DU bleeding due to rupture. This is the first detailed case series of massive DU bleeding as a warning signal of ruptured HAP after LDLT. When recipients present with hematemesis or tarry stool, HAP should be included in the differential diagnosis.

## Data Availability

Not applicable.

## Abbreviations

DU: Duodenal ulcer
HAP: Hepatic artery pseudoaneurysm
LDLT: Living donor liver transplantation
POD: Postoperative day
PUD: Peptic ulcer disease
RHA: Right hepatic artery
TAE: Transcatheter arterial coil embolization
